# The Role of EFL Teachers' Self-Efficacy and Emotional Resilience in Appraisal of Learners' Success

**DOI:** 10.3389/fpsyg.2021.817388

**Published:** 2022-01-04

**Authors:** Yuxiu Xue

**Affiliations:** School of Foreign Languages, Yancheng Institute of Technology, Yancheng, China

**Keywords:** emotional resilience, teachers' self-efficacy, teacher resilience, pedagogical implications, learner's success

## Abstract

Different elements in education should be taken into account in the development of education which affects learners' success. Educators are one of the main elements of any educational program, primarily in mainstream education, and there is considerable research recognizing this and the fact that educators have a significant effect on learners' success. Therefore, education can be enhanced simply by enhancing educators' effectiveness. Moreover, because of the importance of educators' factors, many researchers have emphasized educator attributes over the last 20 years. In an attempt to better explain the interaction between educator-related concepts in the setting of English as a Foreign Language (EFL), educator self-efficacy has a significant impact on the educators' everyday lives as well as on their learners and is regarded as an important factor in successful education and instruction. Furthermore, as one of the characteristics, teacher resilience is a multifaceted and developmental concept that has newly captured the interest of some scholars, specifically in the last 20 years, allowing educators not only to face difficult situations to survive but also to recover and prosper. To this end, the current review tries to emphasize these two significant factors in regulating learners' success. Corresponding to this review, some suggestions for further research are provided and pedagogical implications are proposed.

## Introduction

The degree to which learners have attained an instructional objective is known as learners' success. It is the students' academic performance as an educational result, including the information, abilities, and notions obtained and maintained in the course of learning within and outside the learning environment (Hwang et al., 2017). A learner's success can be said to be the fruitful achievement of a scholastic task at any prearranged level that relies on several interlinked elements and cannot be ascribed to solely a single element (Paat et al., 2020). On the one hand, assets are the constructive elements that exist in a person, like self-efficacy, ability, and management. On the other hand, resources are the outer elements, like guardians' support, educational setting, and the community (Anakwe and Dikko, 2018). Among the external elements, studies have particularly highlighted the outstanding function of people working as educators, managers, and advisers in assisting individuals to improve their scholastic practices and success (Ahmed et al., 2017; Wang and Guan, 2020). Generally, educators must be regarded as crucial characters among the different shareholders in an academic framework that can influence the success or failure of learners and this framework as a whole (Derakhshan et al., 2020) and particularly the role of foreign language educators can be harder since they need to instruct a language they are not native in Shirazizadeh et al. (2019) and Fathi et al. (2021). Educators are being emphasized in the field of education and learning because when they are in emotional equilibrium and well-adapted, learners will be impacted constructively, thereby enhancing the educational cycle, which leads to students' success (Yang, 2021). Additionally, many of these elements influence EFL learners' educational and teaching cycles. For instance, sadness, stress, and anxiety greatly indicate a decline in scholastic performance because learners who report extreme symptoms of mental distress are less likely to claim the scholastic achievement (Ahmed and Julius, 2015; Wang and Guan, 2020). Nonetheless, an important section of the studies on educator-related elements has explored the influences of educator relational elements on learners' success; hence, the function of educators' issues like self-efficacy as possible backgrounds of learners' success has endured subtle in language learning investigations (Lu and Mustafa, 2021). It is believed that educators' capability of controlling time, space, tasks, contents, social relationships, and learners' behavior is an element that influences educator self-efficacy and can impact scholastic performance in the long run (Ahmed and Julius, 2015). Language students are instructed and led by educators who need to have sufficient training and knowledge to offer students needed information by executing proper educational abilities and strategies (Han, 2021a; Xie and Derakhshan, 2021). Supporting maintained education of foreign languages has the requirement of educators having a great degree of self-efficacy when completing instructional activities to assist with education (Bao et al., 2021). Learners' success depends primarily on a variety of elements, including educator efficiency (Liu et al., 2020). The most important individual resource available to educators is self-efficacy which refers to a person's awareness of his/her capacities for achievement (Fathi et al., 2020).

Self-efficacy, or conviction in the ability to accomplish a task, is a strong indicator of success in different scholastic environments (Schunk and Dibenedetto, 2016). Educators' self-efficacy alludes to the educators' conviction in individual ability to carry out responsibilities in the class (Usher, 2015). Educator self-efficacy can enhance education in a way that educators develop educational techniques to support their learners cognitively, socially, and emotionally (Chu, 2011). People with high levels of self-efficacy believe that they have the strategic capabilities, abilities, and information needed to perform educational tasks; as a result, educators with a great degree of self-efficacy can spend more time helping learners who are encountering difficulties in class and commending them for their scholastic success (Chesnut and Cullen, 2014).

Recent studies have emphasized specific psychological concepts that discourage language education and lead to constructive language-learning practices with new attention being paid to the resilience of language-learning in this area (Kim et al., 2017). Indeed, resilience is another significant character attribute associated with learners' success. This alludes to the capability of enduring challenges and recovering and the capability of being impervious to and resilient in the face of diverse stressors appears to be crucial for educators who are regularly overworked (Ayoobiyan and Rashidi, 2021). Furthermore, it is one of the characteristics that closely pertain to the rapidly developing area of educator efficacy (Gu and Day, 2013). Resilience is a concept in the positive psychology literature that emphasizes organizations' and individuals' strong points and self-control to adjust to unforeseen situations (Cooke et al., 2016; Wang et al., 2021).

Resilient entrepreneurs who show a great level of being unbiased and adjust quickly to variation are well prepared for success (Martinelli et al., 2018; Xue, 2021). In addition, educator resilience can be characterized as the ability of educators to recover despite hardship and unpredictable situations encountered every day (Gu and Day, 2013). Moreover, the resilience of educators is explained as a dynamic cycle in which their specific attributes are associated with situational resources to characterize their reactions to unfavorable occurrences (Mansfield et al., 2012). The emotional reaction to everyday classroom encounters, emotional control, and dealing with anxiety is known as emotional resilience (Xue, 2021). It involves individual characteristics, attributes, and/or techniques that educators use in dealing with challenges like the capability of controlling their feelings and not taking matters personally, possessing humor, being emotionally smart, taking pleasure in instructing, and possessing a sense of individual satisfaction (Mansfield et al., 2012). Indeed, emotional resilience is regarded as a dynamic cycle that includes active association and debate between a person and his or her setting (Tait, 2008). Some scholars have discovered that resilient educators are those who obtain fulfillment from their careers, react constructively to demanding in-class circumstances, and show successful techniques for managing hard circumstances and/or learners (Tait, 2008). On the one hand, some researchers consider an educator's emotional resilience as a personal characteristic and characterize it as the trait or ability of an educator to recover after having encountered possible risks (Brunetti, 2006). On the other hand, others characterize it as a result of utilizing stamina constructively when encountering difficult circumstances (Patterson et al., 2004). In addition, resilient educators show pride and satisfaction, have behavioral administration abilities, control deconstructive feelings, and identify with difficult learners (Howard and Johnson, 2004).

A noteworthy share of research on teacher-related issues has examined the role of educator relational elements on learners' success. For instance, a large body of these research identified teachers' psychological and individual factors to be dominant in the process of language learning, including teachers' emotional intelligence, self-efficacy, resilience; emotion regulation (Brackett et al., 2010; Seifalian and Derakhshan, 2018; Fiorilli et al., 2019; Fathi and Saeedian, 2020; Fathi et al., 2021; Han, 2021b; Han and Wang, 2021). However, regardless of the range of research efforts, to the best of the researcher's awareness, there is not a comprehensive review that concurrently considers the relations between EFL teachers' self-efficacy along with emotional resilience and their effective functions on learners' success. Because of the positive role of self-efficacy on diverse extents of teacher presentation, and as an attempt to clarify this research gap, the current review is an attempt to consider the powerful role of teachers' self-efficacy and resilience in encouraging success among learners in the EFL context.

## Review of Literature

### Emotional Resilience

Positive psychology studies have viewed the concept of resilience as being a dynamic and environmentally-related concept, rather than as a static trait in a person's history, and have connected it to ideas like “well-being” and “flourishing” (Fredrickson, 2001). Furthermore, the manners in which social situations impact competence for resilience have been maintained by socio-cultural studies (Oswald et al., 2003). Resilience is molded by a wider range of situational attributes that dynamically interconnect to offer risk (testing) or protective (favorable) elements (Wosnitza et al., 2018). Resilience alludes to the cycle of ability or result of an effective adjustment despite difficult situations (Luthar et al., 2000; Bobek, 2002). Educator resilience has also been characterized as a trait that allows educators to sustain their dedication to education and instructional practices in the face of difficult situations and repeated setbacks (Brunetti, 2006). It appears that educators need resilience as a characteristic to drive development to be confident and effective in class. In particular, educator resilience is characterized as a cycle or result of constructive adjustment and growth in difficult situations and settings that can be created by people or dynamically integrated surrounding elements (Ayoobiyan and Rashidi, 2021). The notion of resilience is found in the discourse on education as an emotional practice. Feelings are an aspect of resilience since educators adopt techniques to sustain their dedication and well-being (Beltman and Poulton, 2019). At the same time, feelings play an important role in creating and maintaining resilience. Above all, constructive feelings enhance and advance resilience. Furthermore, resilience can play an integral role in the cycle of emotional experiences. Concerning these intricately woven concepts, the ability to be emotionally resilient is crucial for scholars since it is with the help of this ability that they sustain a unified expert individuality (Yang et al., 2021). Educator resilience alludes to the cycle of, competence in, and result of constructive adjustment and continuous expert dedication and development when encountering difficult situations.

Resilient educators have the inclination of reacting constructively in demanding classrooms and schools, show successful techniques for cooperating with demanding learners and increase work fulfillment (Tait, 2008). One must highlight the cycle of growing resilience as opposed to distinguishing risks and protective elements that may vary by person and circumstance for the sake of comprehending resilience (Gu and Day, 2013). Ever since, the study of resilience has shifted from distinguishing protective elements that lead to adjustable results to comprehending the cycle of arranging and conquering difficulties and creating actual techniques (Castro et al., 2010). There have been studies on resilience for decades in the fields of developmental psychology and psychopathology to comprehend high-risk groups, specifically at-risk adolescents (Goldstein and Brooks, 2006; Wright and Masten, 2006). Scholars started to concentrate more on educator resilience in the field of educator training to better comprehend the growth of educator identity (Kirk and Wall, 2010; Wang and Guan, 2020), work fulfillment and inspiration (Brunetti, 2006; Kitching et al., 2009), educator burnout and anxiety (Howard and Johnson, 2004), choices regarding professions, and the effectiveness of education (Gu and Day, 2013).

Mansfield et al. (2012) developed four main streams of educator resilience, including professional, emotional, social, and motivational elements. The professional dimension is related to the ability to consistently run a qualified education and learning company. In the context of education, the vocational aspect consists of educational ability and capacity, institution, arrangement, facilitating successful education, class administration, and flexibility. Educators must be able to demonstrate a higher level of self-confidence, inspiration, and perseverance when coping with a large number of work problems through the emotional dimensions. The social dimension explains educators' relational abilities in finding help, cooperative networking, and continuous expert growth (Lodari and Sabaruddin, 2018). Finally, the personal dimension primarily involves self-efficacy, the integration of inner and outer abilities, and simultaneously reconstructs both the success and resilience of education and learning.

### Teachers' Self-Efficacy

The social cognitive theory offers a system for anticipating practice and it is a theory of education and alteration, and a process of self-awareness. As stated by Bandura (2011) people are neither automatically managed by outer occurrences nor triggered by internal pressures. Rather, they actively take part in and influence their personal growth, adjust to variations and changes, and gradually begin self-reestablishment. Therefore, this theory allows educators to comprehend their demeanors and convictions, which gives them insight and understanding of their convictions regarding their educational abilities (Bandura, 2011). His theory is a conceptual model that embraces the root or origin of efficacy convictions, their format and role, the cycles through which they result in various impacts, and the potential for alteration (Brouwers and Tomic, 2000). It is a hypothesis that demonstrates how the intellectual, behavioral, and surrounding factors of human conduct connect and influence one's convictions about their ability to create impacts (Bandura, 2011; Han and Wang, 2021).

Being inspired by one's will and convictions, self-efficacy is postulated as an inspirational concept which comes from social cognitive theory and is dependent on self-awareness of capacity as opposed to an individuals' actual capacity degree that is a prevailing subject in describing how a person accomplishes, ponders, and responds in case of inspiring conditions (Tschannen-Moran and Hoy, 2007). How people regard their capability of succeeding in a particular task is known as self-efficacy. How people fully understand their potential for efficacy affects to a great degree the way they deal with and carry out tasks, goals, or difficulties, and how much they endeavor in their actions, how patient they are during hard times, how they face issues, and the degree of anxiety they experience in compromising circumstances (Bandura, 2011). Sometimes referred to as educational efficiency, educator efficiency alludes to the educators' conviction that they can affect learners' educational results. It is regarded as an inspirational concept that is future-directed and reflects the educators' convictions about the ability to instruct assignments (Tajeddin and Khodaverdi, 2011).

A teacher's conviction in their ability to help learners improve their scholastic performance is known as educator's self-efficacy (Mok and Moore, 2019). It is defined as educators' individual beliefs regarding their capacity to carry out scholastic duties and educators' self-efficacy is a group of convictions that an educator has about the capability and capacity to instruct and affect a learner's practice and performance, despite external forces or impediments (Klassen et al., 2014). Specifically, an educator with self-efficacy is the person who has faith in himself and his expert skills, and an educator with a high level of efficacy is responsible for the learners' education and may regard learners' setbacks as a thrust to make greater endeavors to enhance performance. These educators spend more time overseeing and cooperating with learners and offering a means of increasing learners' involvement. Efficacious educators are more inclined to not only cover the syllabus but to also use educational techniques that improve learners' education (Sharma and George, 2016). They take more chances; have greater expectations of themselves and their learners, resulting in greater scholastic benefits among students. Educators who do not possess efficacy are proved to demonstrate weaker dedication to instruction, put in less time for subject matters in their domains of discerned inefficacy, and dedicate less overall time to scholastic matters (Akbari et al., 2008). Additionally, educators with a great level of efficacy are usually inclined to show a higher level of arrangement and management. Moreover, they are more receptive to new notions and are inclined to try new strategies and techniques to meet learners' demands in a better way. Efficacy convictions affect educators' perseverance when things are not going well and make the educator more resilient when setbacks are encountered (Tschannen-Moran and Hoy, 2007). Educators with greater efficacy demonstrate greater excitement for and inclination toward instruction, are more dedicated, and will more likely remain in the profession (Akbari et al., 2008). As a result, educator self-efficacy has proven to be a multidimensional concept affected by many sub-competencies, reliant on the teaching circumstances (Sharma and George, 2016). As Tschannen-Moran and Hoy (2007) stated, teacher efficacy as a concept that impacts educators' perseverance and educational performance, learners' success, and teachers' opinions that they can assist the most unenthusiastic learners to learn.

According to Bandura (2011), a person's self-efficacy can be built from four major origins: proficiency experience, secondary experience, oral or social convincing, and arousal or emotional condition. The proficiency experience is self-attained through direct experience or self-recognition learning, by completing courses, seeking information and advice from professionals. As stated by Tschannen-Moran and Hoy (2007), mastery or positive experiences are the fulfillment achieved through the success of previous instruction. The result of observing others carrying out the activity is a secondary experience. However, vicarious experience is the consequence of inspecting others' performance during performing a task. The way individuals look for competent and decent frameworks, based on which they can shape themselves, is known as secondary experiences offered by social frameworks. They familiarize themselves with these models, and if observed, they can strengthen their convictions in their ability to triumph. Social convincing is another origin for impact and it orally enhances self-efficacy, leading to the growth of abilities and greater individual efficacy. It is an oral convincing that makes one have faith in the fact that they can accomplish their objectives if they utilize their capacities and clear their doubts (Tschannen-Moran and Hoy, 2007). Oral or social convincing alludes to the motivation or demotivation of situational and social agents like managers, educators, guardians, companions, and peers. The last origin is related to conditions created by emotional and psychological factors like enthusiasm, anxiety, and stress, which tests one's emotions and influence their emotional condition (Baharloo and Mehrpour, 2016).

## Implications and Future Directions

This review makes some serious assistance to the present body of literature. Educators, their educators, and language policymakers should discover the worth of teachers' positive function such as their resilience and self-efficacy for influential instruction that consequently brings about the learners' success.

In learning a foreign language, inspiration, endeavor, and success can be increased by establishing constructive feelings and reducing deconstructive ones on both the side of the educator and the student. Increasing resilience may help EFL educators become better experts and sympathetic teachers who support learners' educational progress (Gu et al., 2015). The notion of resilience turns EFL educators into better experts, makes them more committed and inspired, and increases their perseverance. They end up being always ready to leave various beneficial effects on their teaching and learning projects. Constructive results connected to resilience include mitigating the deconstructive influences of anxiety, facilitating adjustment, and developing successful coping abilities to address variation and hardship that lead to learners' success. Various scholars and researchers are beginning to explore resilience as an internal trait to help educators cope with the stressors they face in their careers and to not only survive but also succeed at school (Gloria et al., 2013).

Self-efficacy is the explanation a person gives for their achievements and performance, and it is created by present self-convictions and directly influences one's inspiration to succeed in future circumstances. Therefore, educator training programs can be developed to constructively increase educators' consciousness of their possible inefficacies and a greater degree of educator self-efficacy. As opposed to educators with low self-efficacy convictions, educators with high levels of self-efficacy convictions are more inclined to use didactic creativity in their lessons and to utilize class administration techniques and suitable instructional strategies that inspire learners' independence and lessen custodial management, to be responsible for learners with special educational demands, to deal with class issues, and to ensure learners complete their assignments (Eghtesadi and Jeddi, 2019). Educators with great degrees of self-efficacy will go beyond their present capacity level and make use of the time and stamina required to arrange and employ new techniques since they are certain of possessing control over learners' educational results (Hattie and Zierer, 2018). Educators with great degrees of self-efficacy have more resilience and are ready to take chances by employing new strategies with learners since they possess the conviction that they can effectively increase learners' success (Malanson et al., 2014). This review can assist with restructuring suitable educator training programs that enhance the comprehension of EFL educators' self-efficacy convictions and their significance in education by studying the relevant efficacy hypotheses, sources, and elements of EFL educators' self-efficacy.

Correspondingly, EFL teachers should benefit from the results of the study since it is of prominence for them to increase their competence, teaching potentials, abilities, and assertiveness to cause universal effects for their precise language learning environments. In addition, the EFL educator training program must pay more attention to practical techniques to improve EFL educators' efficacy convictions, emotional abilities, and contemplation skills for pre-service EFL educators. Moreover, managers and educator trainers are encouraged to acquaint educators with self-efficacy strategies to conquer the numerous hardships involving their careers. For instance, workshops must be provided where educators can work together in a constructive manner, which has been proposed to be a successful method for improving educator efficacy, ultimately prompting learners' success. Educators with high levels of self-efficacy are good at preparing and organizing classes, enthusiastic about and dedicated to their career, more focused on students' demands, more embracing of new notions, persevere better in their profession, and deal with difficult students better (Tschannen-Moran and Hoy, 2007).

The outcomes of the research may help foreign language instruction policy creators in general and language organization administrators in specific to improve language educators' consciousness of the importance of essential elements like resilience and efficacy in the educational cycle. School organizations should promote and cultivate EFL teachers' resilience at the beginning of their qualified occupations to preserve their assurance, commitment, enthusiasm, persistence, and professional fulfillment in an enduring course that results in success in students. Furthermore, a caring and helpful atmosphere should be provided by school managers since it is stated that resilience is a construct that can be taught and cultivated by a protective and sympathetic milieu where educators can improve a more resilient and truthful individuality (Howard and Johnson, 2004; Castro et al., 2010). Educator training programs help pre-and in-service educators become conscious of successful educator traits like resilience and efficacy if the goal is to train successful educators who can eventually prompt learners' success. So by developing teachers' self-efficacy and resilience, school members can guarantee the learners' success.

The study also provides psychological implications for curriculum creators to not disregard the function of educators in curriculum design. Activities and assignments that emphasize the function of educators and promote their resilience need to be designed. Moreover, further study can be carried out to distinguish specific techniques that can be adopted by resilient EFL educators, like seeking support, problem-solving, dealing with difficult connections, seeking restoration, sustaining a career-life equilibrium, taking part in continuous career growth, establishing emotional borders, and making use of humor (Doney, 2013). The present review provides some empirical proof that educator self-efficacy and resilience may be associated with students' success, thus it supports future studies to further explore these two characteristics for educators and students together. It cannot be expected that educators, non-native ones in specific, can manage English classes without receiving specific training. Future researchers are encouraged to be conducted a more experimental investigation concerning the reciprocal interactions among EFL teachers' self-efficacy, resilience, to locate these outcomes into EFL settings.

## Author Contributions

The author confirms being the sole contributor of this work and has approved it for publication.

## Conflict of Interest

The author declares that the research was conducted in the absence of any commercial or financial relationships that could be construed as a potential conflict of interest.

## Publisher's Note

All claims expressed in this article are solely those of the authors and do not necessarily represent those of their affiliated organizations, or those of the publisher, the editors and the reviewers. Any product that may be evaluated in this article, or claim that may be made by its manufacturer, is not guaranteed or endorsed by the publisher.
